# Multiple Sequence Alignments Enhance Boundary Definition of RNA Structures

**DOI:** 10.3390/genes9120604

**Published:** 2018-12-04

**Authors:** Radhakrishnan Sabarinathan, Christian Anthon, Jan Gorodkin, Stefan E. Seemann

**Affiliations:** 1 Center for Non-Coding RNA in Technology and Health, Department of Veterinary and Animal Sciences, University of Copenhagen, Grønnegårdsvej 3, 1870 Frederiksberg C, Denmark; sabari@rth.dk (R.S.); anthon@rth.dk (C.A.); gorodkin@rth.dk (J.G.); 2 National Centre for Biological Sciences, Tata Institute for Fundamental Research, Bangalore 560065, India

**Keywords:** RNA secondary structure, RNA structure boundary, RNA domain, non-coding RNA gene finder

## Abstract

Self-contained structured domains of RNA sequences have often distinct molecular functions. Determining the boundaries of structured domains of a non-coding RNA (ncRNA) is needed for many ncRNA gene finder programs that predict RNA secondary structures in aligned genomes because these methods do not necessarily provide precise information about the boundaries or the location of the RNA structure inside the predicted ncRNA. Even without having a structure prediction, it is of interest to search for structured domains, such as for finding common RNA motifs in RNA-protein binding assays. The precise definition of the boundaries are essential for downstream analyses such as RNA structure modelling, e.g., through covariance models, and RNA structure clustering for the search of common motifs. Such efforts have so far been focused on single sequences, thus here we present a comparison for boundary definition between single sequence and multiple sequence alignments. We also present a novel approach, named RNAbound, for finding the boundaries that are based on probabilities of evolutionarily conserved base pairings. We tested the performance of two different methods on a limited number of Rfam families using the annotated structured RNA regions in the human genome and their multiple sequence alignments created from 14 species. The results show that multiple sequence alignments improve the boundary prediction for branched structures compared to single sequences independent of the chosen method. The actual performance of the two methods differs on single hairpin structures and branched structures. For the RNA families with branched structures, including transfer RNA (tRNA) and small nucleolar RNAs (snoRNAs), RNAbound improves the boundary predictions using multiple sequence alignments to median differences of −6 and −11.5 nucleotides (nts) for left and right boundary, respectively (window size of 200 nts).

## 1. Introduction

The function of RNA is often guided by its structural conformation, which is in turn determined by its sequence composition. The classic examples of structured non-protein coding RNAs (ncRNA) include transfer RNAs (tRNA), ribosomal RNAs (rRNA), and precursor microRNA (pre-miRNA). In addition, untranslated and intronic regions of the messenger RNAs (mRNA) often contain *cis*-regulatory structures, e.g., riboswitches, iron response elements (IRE), internal ribosome entry sites (IRES), and selenocysteine insertion sequences (SECIS). Furthermore, long non-coding RNAs (lncRNAs) can contain local functional structures, e.g., lncRNA *GAS5* forms a secondary structure that binds the glucocorticoid receptor (GR), which in turn prevents GR from binding to the glucocorticoid-response elements contained in responsive genes [1]. Moreover, mRNAs and lncRNAs are regulated by a plethora of molecules binding to it and these can influence its global conformation, where their local structures are often sufficient for recognition by RNA binding proteins and are probably more relevant for regulatory function.

Defining the RNA structure domains has been addressed at the single sequence level, first explicitly by Dotu et al. [2]. They described a fitness function for all segmentations of subwords of a sequence based on the base pairing probability matrix. These matrices are usually calculated from the respective sequence by McCaskill’s partition function approach [3]. Their fitness function calculates the difference between the base pairing probabilities inside of a segment ($P_{I}^{bp}$) and probabilities of pairing between nucleotides inside the segment and nucleotides outside ($P_{O}^{bp}$) normalized by the length of the segment. Weight factors for the two terms in this fitness function had been optimized for microRNAs (RNA structures of a single hairpin loop). We used a similar concept upon searching for the local region that is most disrupted as a result by single nucleotide polymorphisms [4] and further to optimize borders of an RNA structure under constraints [5]. For example, if the structure of the single sequence is taken from the context of a multiple structure alignment, it is of interest to find the exact boundaries of its minimum free energy folding. As a first attempt for finding RNA structure boundaries from multiple structure-based alignments, Will et al. had introduced structure-based alignment reliabilities (STARs) in LocARNA-P that calculate columnwise and global reliabilities of alignments based on sequence and structure similarity [6]. The usefulness of the approach had been shown through the boundary refinement of RNAz predictions in Drosophila.

The structures of most ncRNAs and *cis*-regulatory elements are evolutionarily conserved independently of their sequence conservation. Thus, many RNA folding methods utilize the conservation signal, such as compensating base pair changes, for predicting conserved RNA secondary structures and structured ncRNAs from a set of aligned or un-aligned multiple sequences. We recently showed that there is indeed an effect for clustering RNA structures, e.g., from the same RNA family, when going from single sequences to multiple structure-based RNA alignments containing covariating base pairs [7]. Here, we address, in a systematic fashion, whether the search for RNA structure boundaries can be improved using multiple aligned sequences compared to single sequences. Our method, named RNAbound, extends on the fitness function of Dotu et al. by also considering base pairing probabilities that span over the structure boundaries. Base pairing probabilities are calculated with PETfold [8] whose model of unified evolutionary and thermodynamic information had been shown to perform well for predicting the consensus structure from a set of aligned sequences [9].

Genomic screens for ncRNAs predict thousands of potential genomic loci of structured RNAs, such as CMfinder [10], Evofold [11] and RNAz [12,13] (reviewed in [14]). Since the genomic sequences are exceptionally long, the abovesaid programs either predict local structures (e.g., CMfinder) or slice the genomic sequence into overlapping windows and search for structured ncRNAs within each window individually. Although windowing helps to reduce the computation time, the fixed window size may be larger or shorter than the actual length of the structured RNAs. For this reason, the further analysis of these data requires highly accurate prediction of RNA structure boundaries.

Here, we investigate into putative improvements of structure boundary predictions when traversing from single sequences to multiple sequence alignments. We also present RNAbound, a new software that implements a novel fitness function for searching boundaries of structured RNA regions from the base pairing probability matrix.

## 2. Materials and Methods

### 2.1. Fitness Function for Boundary Detection

In this section, we introduce a new fitness function to find the segment [k,l] with the highest mass of *self-contained* base pairings inside of the larger sequence [1,n], with 1+f≤k<l≤n−f where *f* is the length of flanking sequences up- and downstream of segment [k,l]. The self-containedness of the secondary RNA structure of a segment [k,l] is defined as the base pairs inside [k,l] for which no base pairing exist to bases outside [k,l] and no base pairs exist that span over [k,l] within a margin around [k,l]. The margin is defined as the sequences [k−f,k−1] and [l+1,l+f] with appropriate sequence length *f*. The three components can be described by scores based on base pairing probabilities: (1) score $I^{bp}$ is the geometric mean of paired probabilities between bases inside [k,l]; (2) score $O^{\neg bp}$ is the geometric mean of unpaired probabilities of bases inside [k,l] to bases outside of [k,l]; and (3) score $S^{\neg bp}$ is the geometric mean of unpaired probabilities of bases in the flanking sequence [k−f,k−1] (upstream of [k,l]) to bases in the flanking sequence [l+1,l+f] (downstream of [k,l]). The geometric mean has been applied for sequence length normalization because it is not biased to a few high probabilities when calculating averages of probabilities that are exponential distributed, and the Boltzman distribution of base pairings is exponential. Finally, the fitness function $F_{kl}$ is defined as the product of the three normalized scores:

(1) $$\begin{array}{ccc} F_{kl} & = & I^{bp}\times O^{\neg bp}\times S^{\neg bp}\\ & = & {\left[\prod_{m=k}^{l}\sum_{n=k}^{l}p_{mn}\right]}^{\frac{1}{l-k+1}}\\ & & \times {\left[\prod_{m=k}^{l}\left(1-\sum_{n=[1,k),(l,N)}p_{mn}\right)\right]}^{\frac{1}{l-k+1}}\\ & & \times {\left[\prod_{m=k-f}^{k-1}\left(1-\sum_{n=l+1}^{l+f}p_{mn}\right)\right]}^{\frac{1}{f}}, \end{array}$$

where $p_{mn}$ is the base pairing probability between position *m* and *n*. To avoid our boundary estimates from being mostly driven by a small number of high base bairing probabilities, we replace the base pairing probabilities $p_{mn}$ with base pair scores. The base pair score, similar to the implementation in LocARNA-P [6], is defined as $max\left(0,\log\frac{p_{mn}}{p_{0}}/\log\frac{1}{p_{0}}\right)$. The log odds score $\log\frac{p_{mn}}{p_{0}}$ describes a specific base pairing against the null model of a random pairing. We normalize with $\log\frac{1}{p_{0}}$ to transform the base pair score to a maximum of 1. As a minimum value for the base pair score, we chose zero as in the case where $p_{mn}$ is equal to the random pairing ($p_{0}$). We implement the fitness function by replacing the product of probabilities with the sum of log probabilities.

Finally, the position of the segment detected with maximum score $F_{kl}$ is reported as the boundaries [u,v] of structured region:

(2) $$\left[u,v\right]=\underset{k,l}{\mathrm{argmax}}F_{kl}.$$

Our implementation, named RNAbound, accepts as input a single sequence or a multiple sequence alignment. The base pair probabilities $p_{ij}$ are calculated for a single sequence with RNAfold v2.4.1 [15] (with *−p* and other default parameters), and for a multiple alignment with PETfold v2.1 [8] (with *−r* and other default parameters).

### 2.2. Benchmark Data Set

We defined a set of annotated structured regions (*n* = 3103) by using the known structured RNA sequences from various resources such as Rfam (v12.2) [16], mirBase (v21) [17], tRNAdb [18], rRNA (silva) [19] and snoRNAdb [20]; and mapped them into the human genome (hg38). In case of sequences from Rfam seed alignments, we used BLAST [21] tool to find 100% identical match in the human genome. Furthermore, for each structured region, we generated a multiple sequence alignment with homologous sequences of other vertebrate organisms. We selected a representative subset of the organisms present in the 100way UCSC alignment [22] based on the phylogeny and sequence coverage: Chinchilla lanigera (chiLan1), Dasypus novemcinctus (dasNov3), Felis catus (felCat8), naked mole-rat genome (hetGla2), Jaculus jaculus (jacJac1), Microtus ochrogaster (micOch1), Mus musculus (mm10), Killer whale (orcOrc1), Oryctolagus cuniculus (oryCun2), Black flying-fox (pteAle1), Ictidomys tridecemlineatus (speTri2), Trichechus manatus latirostris (triMan1), and Chinese tree shrew (tupChi1). We used multiz (tab) [23] to form a multiple sequence alignment, by keeping the human sequence as reference, and performed pairwise alignments with the listed organisms. In the pairwise alignment step, we only considered alignments if (a) the human annotation (plus 20 nts flanking to each side) is included in the pairwise alignment, and, (b) for shorter annotations, a window of at least 200 nts centered on the annotation is included in the pairwise alignment.

Furthermore, we created windows of sizes 100, 150, and 200 nts that are centered around the middle position of the structured RNA annotations and performed the following quality filters: (a) the selected window size of an alignment should cover the start and end of the mapped known RNA sequence plus flanking sequences of 10 nts, (b) no overlapping annotations within 200 nts upstream or downstream, (c) the number of bases involved in the base pairing (of the annotated secondary structure obtained from Rfam) should be greater than or equal to 20%, (d) sequences with gaps more than 75% of the length of the alignment are removed from the alignment, (e) after gap filtering the alignment should have minimum three sequences, and (f) the mean pairwise sequence identity of the alignment should be ≥60% and <95%. The numbers of filtered structured RNA annotations in each step are listed in Supplementary Table S1. The remaining numbers of alignments after filtering and their corresponding RNA families are shown in Table 1.

Under each Rfam family, we further classified the structures into two main groups: single hairpin structure and branched structures based on the abstract shape (predicted using RNAshape [24]) of the consensus secondary structure obtained from Rfam. The single hairpin structure group covers miRNAs, short cis-regulatory RNAs, and C/D box small nucleolar RNAs (snoRNAs), whereas the branched structures group covers the rest including tRNAs, snoRNAs such as H/ACA box and scaRNAs, small nuclear RNAs (snRNAs) and ribozyme.

### 2.3. Benchmarking

In order to test the performance of RNAbound, we used the benchmark dataset of multiple sequence alignments of the window sizes 100, 150, and 200 nts centred around structured RNA regions (see Table 1). For each window category, we ran RNAbound with PETfold on the alignments to predict the boundaries of the self-contained structured regions. Similarly, we ran RNAbound with RNAfold on the human sequence (without gaps) from each of the alignments under the different window sizes. The parameters of the fitness function $F_{kl}$ are set to f=10 and $p_{0}=0.0005$. The *boundary difference* between the RNAbound predicted boundaries ([u,v]) and the actual boundaries of structured regions was computed as

(3) $$\begin{array}{c} left=\mathrm{actual}\;\mathrm{start}-\mathrm{predicted}\;\mathrm{start}(u),\\ right=\mathrm{predicted}\;\mathrm{end}(v)-\mathrm{actual}\;\mathrm{end}. \end{array}$$

If the value of left or right boundary difference is zero, then the predicted boundaries are equal to the actual boundaries, whereas, if the values are positive, then the predicted start or end position is outside the actual start or end position (i.e., excess from the actual boundaries), and the negative value indicates the predicted boundaries are inside the actual boundaries (i.e., short of the actual boundaries). We are not presenting absolute differences, such as the sum of the absolute value of the left and right boundary difference, because information will be lost about which boundary (left or right) has better or poor performance and whether the predicted boundary is in excess or short of the actual boundary.

Furthermore, to compare our results with the Dotu et al. approach [2], we implemented their fitness function (Equation (4)) and predicted structure boundaries with the recommended weight combination of $w_{1}=2$ and $w_{2}=1$ and base pair probabilities computed with PETfold or RNAfold

(4) $$\begin{array}{cc} f_{ij} & =\frac{\sum_{i\leq x<y\leq j}w_{1}\cdot p_{xy}-\sum_{x\in [i,j]}\sum_{y\notin [i,j]}w_{2}\cdot p_{xy}}{j-1+1}. \end{array}$$

The difference between the boundaries predicted by the Dotu et al. approach and the actual boundaries was computed using Equation (3). The statistical significance of differences between the predictions by the aforementioned approaches was computed using the two-sided Wilcoxon rank-sum test.

## 3. Results

### 3.1. Boundary Detection of Branched RNA Structures Is Improved with Multiple Sequence Alignment

**Figure 1:**
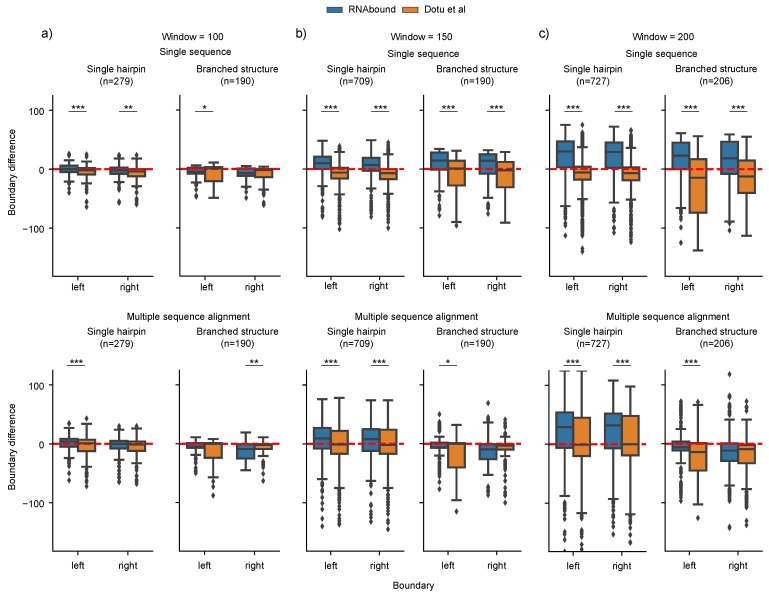
Comparison of actual and predicted boundaries by RNAbound and Dotu et al. on the benchmarking dataset across all families (Table 1) of different window sizes 100 (**a**), 150 (**b**), and 200 (**c**). Under each window category, the top row represents the results obtained from the single sequence and the bottom from multiple sequence alignment. In both cases, we considered structures whose actual base pair start and end positions are within the respective window size. The value on the *y*-axis indicates if the predicted and actual boundaries are the same (if the value is zero), in excess (positive) or short of (negative) compared to the actual boundaries. The distributions that showed significant difference based on the two-sided Wilcoxon rank-sum test are indicated with asterisk symbol (*** *p* < 0.001, ** *p* < 0.01, and * *p* < 0.05).

To determine the ability to predict boundaries of structured RNA in a single sequence versus multiple sequence alignment, we compared the RNAbound predictions with RNAfold and PETfold on the benchmark dataset (see Table 1, see Methods) comprising multiple sequence alignments of different window sizes (100, 150, and 200) centered on the structured RNAs. In the smaller window size 100, where the sequences flanking the actual boundaries are shorter compared to other window sizes, the boundaries predicted are similar for the single and multiple sequence alignment independent of the fitness functions (RNAbound or Dotu et al.) across all Rfam families (Figure 1a, Table 2). For instance, in the case of RNAs that form a single hairpin structure (e.g., miRNAs), the median value of boundary difference for the RNAbound (and Dotu et al. approach) on the single sequence is 0.0 and −2.0 (−2.0 and −4.0) for left and right boundary, and on the multiple sequence alignment it is 4.0 and 0.0 (1.0 and −1.0). In the case of RNAs that form branched structures (e.g., tRNAs and snoRNAs), we observed similar results with the median value of boundary differences of −5.0 and −7.0 (1.0 and −2.0) for left and right boundary on single sequence, and −5.0 and −9.0 (0.0 and −2.0) on the multiple sequence alignment (see Table 2 and Figure 1a).

**Figure 2:**
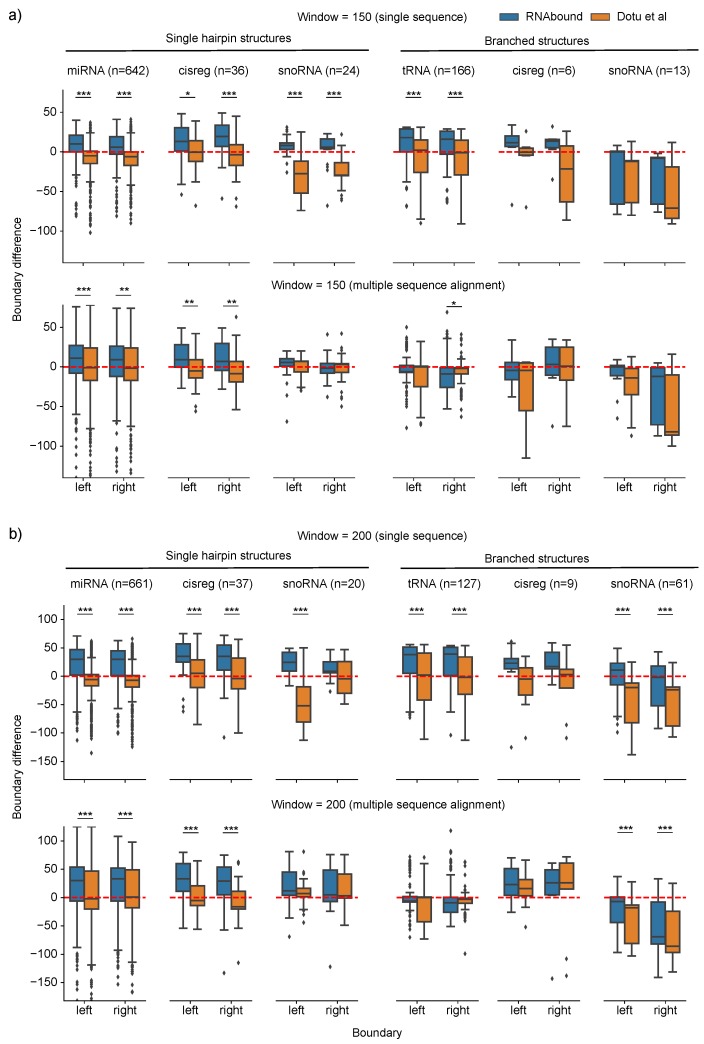
Comparison of actual and predicted boundaries predicted by RNAbound and Dotu et al. on the benchmarking dataset of specific RNA families (Table 1) of different window size 150 (**a**), and 200 (**b**). Under each window category, the top row represents the results obtained from the single sequence and the bottom row from multiple sequence alignment. In both cases, we considered structures whose actual base pair start and end position are within the respective window size. The value on the *y*-axis indicates if the predicted and actual boundaries are the same (if the value is zero), in excess (positive) or short (negative) of the actual boundaries. The distributions that showed significant difference based on the two-sided Wilcoxon rank-sum test are indicated with asterisk symbol (*** *p* < 0.001, ** *p* < 0.01, and * *p* < 0.05).

In the larger window sizes 150 and 200, the boundaries predicted using the single sequence are in excess compared to the multiple sequence alignment for branched RNA structures (see Table 2 and Figure 1b,c). This is particularly evident for the RNAbound predictions on window size 200 with median boundary differences for left and right boundaries of 23.0 and 18.5 on single sequence, and −6.0 and −11.5 on multiple sequence alignment. In the case of the Dotu et al. approach, we observed no such difference between single (−14.5 and −12.5) and multiple sequence alignment (−14 and −9). However, the analysis specific to each Rfam family shows that the impact of multiple sequence alignments for the boundary prediction varies across different Rfam families (Figure 2). In the case of tRNAs, the median boundary difference is close to zero (that is, the predicted and actual boundaries are similar) on multiple sequence alignment and the distribution (interquartile range) of boundaries differences are narrower (centered around zero) compared to the single sequence results, both for RNAbound and Dotu et al. approaches (Figure 2b, Table A1 and Table A2). In other branched structures, such as cis-regulatory RNAs and snoRNAs, the improvement by multiple sequence alignments is varying within the RNA families. For snoRNAs, the left boundaries are better predicted on multiple sequence alignments, but the right boundaries are often better predicted from single sequences (Figure 2b and Table 2). In the window size 200, there are 61 snoRNA branched structures, of which 50 belong to H/ACA-box family snoRNAs that consist of two hairpin structures (adjacent to each other). After looking into the base pairing probability matrices (RNAfold and PETfold) for all these cases, we noticed that, in the case of the single sequence, RNAfold predicts either one long hairpin structure or bifurcated structures with a small stem between 5’ and 3’ ends. In contrast, PETfold predicts two independent stem-loop structures from the multiple alignments. In some cases, both RNAbound and Dotu et al. failed to cover one of these two stem-loop structures within the predicted boundaries, and thus the performance is lower compared to the prediction from a single sequence.

**Figure A1:**
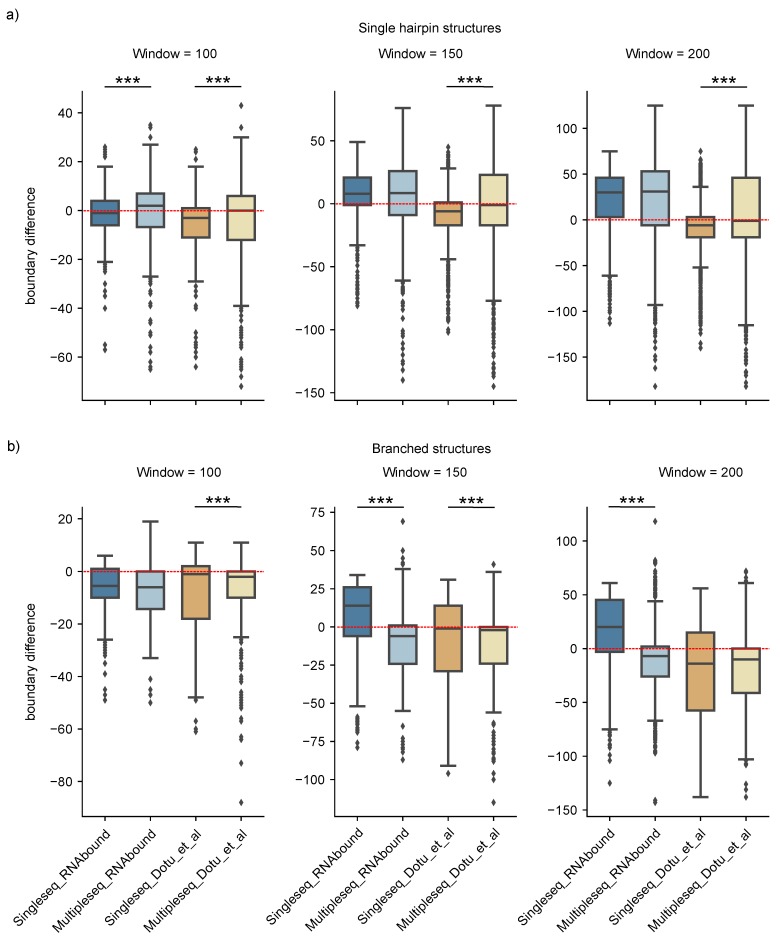
Comparison of RNAbound and Dotu et al. boundary predictions on single sequence versus multiple sequence alignment for all single hairpin structures (**a**) and branched structures (**b**). For simplicity, here we combined the results of left and right boundary differences under each category represented on the *x*-axis. The distributions that showed significant difference based on the two-sided Wilcoxon rank-sum test are indicated with asterisk symbol (*** *p* < 0.001, ** *p* < 0.01, and * *p* < 0.05).

In summary, the benchmarking results show that the structured RNA boundary detection is improved with multiple sequence alignment when compared to the single sequence for the group of branched RNA structures, for both RNAbound and Dotu et al. approaches, but not for single hairpin structures. This improvement is significant with RNAbound for window sizes 150 and 200, and with Dotu et al. for window sizes 100 and 150 (two-sided Wilcoxon rank-sum test *p* < 0.001; see Figure A1). The performance of RNAbound and Dotu et al., however, varies across different Rfam families in the same group.

### 3.2. RNAbound Predictions Are Sensitive to both Single Hairpin and Branched Structures

We next sought to compare the performance of RNAbound and Dotu et al. in predicting boundaries for different RNA families comprising single hairpin and branched structures on the multiple sequence alignment (Figure 2). For miRNAs and cis-regulatory families of single hairpin structures on the window sizes 150 and 200, RNAbound predicted excess boundaries on both the left and right compared to the Dotu et al. approach (Table 2). However, with the Dotu et al. approach, the boundaries predicted are in short compared to the actual boundaries. For snoRNAs that form single hairpin structures, RNAbound performed similarly to the Dotu et al. approach.

**Figure 3:**
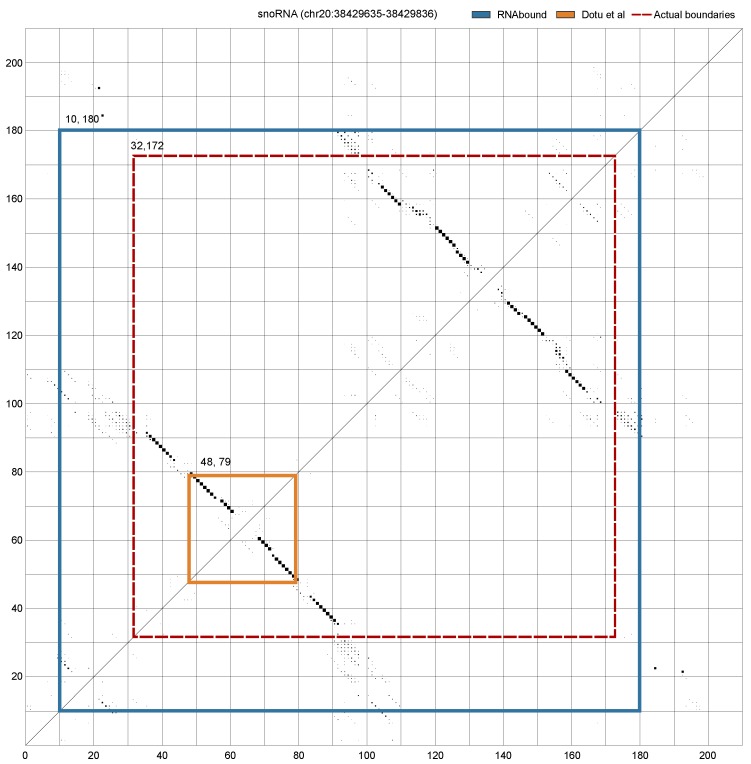
Dot plot shows the PETfold base pairing probabilities computed from the multiple sequence alignment of a window 200 nts centered on an H/ACA-box snoRNA (RF00056). Each dot corresponds to base pairing of two bases in the respective positions (along the *x*- and *y*-axis) and the size of the dot corresponds to the probability. The bigger the size the higher the pairing probability. Due to the gaps in the multiple sequence alignment, the size of dot plot exceeds the window size 200 nts. The actual and predicted boundaries of the snoRNA are highlighted in the plot: (**a**) actual boundaries (31, 172—red dashed lines); (**b**) RNAbound predicted boundaries (10, 180—blue lines); and (**c**) Dotu et al. prediction (48, 79 orange lines). RNAbound predicted an excess of boundaries, 22 bases on the left and eight bases on the right, as compared to actual boundaries. However, the Dotu et al. approach predicted short of boundaries, −16 and −93, respectively, on the left and right boundaries, as compared to actual boundaries.

**Figure 4:**
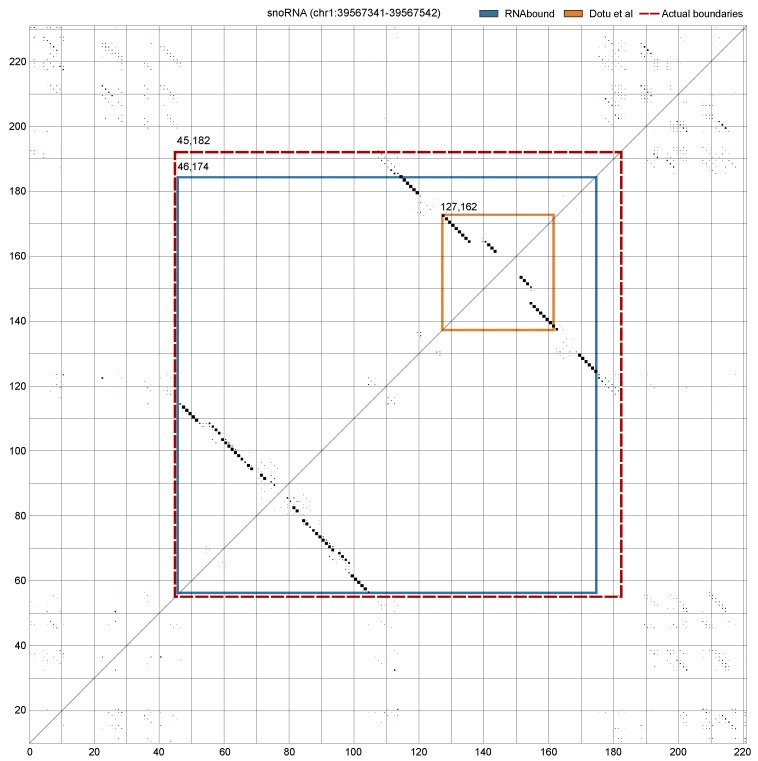
Dot plot shows the PETfold base pairing probabilities computed from the multiple sequence alignment of a window 200 nucleotides (nts) centered on an H/ACA-box snoRNA (RF00431). Each dot corresponds to base pairing of two bases in the respective positions (along the *x*- and *y*-axis) and the size of the dot corresponds to the probability. The bigger the size the higher the pairing probability. Due to the gaps in the multiple sequence alignment, the size of dot plot exceeds the window size 200 nts. The actual and predicted boundaries of the snoRNA are highlighted in the plot: (**a**) actual boundaries (45, 182—red dashed lines); (**b**) RNAbound predicted boundaries (46, 174—blue lines); and (**c**) Dotu et al. prediction (127, 162 orange lines). The boundaries predicted by RNAbound are closer to the actual boundaries (−1 bases short on the left and –8 bases short on the right), whereas the boundaries predicted by the Dotu et al. approach covered only the short substructure and are of −82 and −20 bases short on the left and right, respectively, as compared to the actual boundaries.

The Rfam families that form branched RNA structures show high variability in the boundary prediction for both RNAbound and Dotu et al. approaches on the multiple sequence alignment. In the window size 200, the boundaries predicted for tRNAs are close to zero for both approaches. In the case of cis-regulatory RNAs, the boundaries predicted are in excess for both approaches (Table 2). Interestingly, in the snoRNA branched structures, comprised of H/ACA box and scaRNAs, the boundaries predicted are short compared to actual boundaries for both RNAbound and Dotu et al. approaches. However, the RNAbound predictions are closer to the actual boundaries (with median differences of left and right boundary as −7 and −69) as compared to the Dotu et al. approach (with median differences of −18 and −86). After going through the individual cases of snoRNA results, we noticed that, in some cases, the Dotu et al. approach failed to cover the full snoRNA structure that comprises two hairpins adjacent to each other. Figure 3 shows an example of H/ACA box snoRNA, where the RNAbound predicted boundaries are in excess, 22 bases on the left and eight bases on the right, as compared to the actual boundaries. In contrast, the Dotu et al. approach predicted short of boundaries, −16 and −93 respectively, on the left and right boundaries. In other cases, RNAbound predicted boundaries that are in short of the actual boundaries; however, the boundaries predicted are still closer to the actual boundaries when compared to the Dotu et al. predictions. An example of this is shown in the Figure 4 using the H/ACA box snoRNA. In this case, the shorter boundary prediction of RNAbound can be explained by the exclusively low base pairing probabilities for those bases that are not covered.

In summary, the performance of RNAbound and Dotu et al. approaches are comparable and their predicted boundary differences vary with respect to the RNA families that form either single hairpin or branched secondary structures. Overall, we found that RNAbound predicts boundaries in excess for single hairpin structures, and short for branched structures when compared to actual boundaries on the larger window sizes 150 and 200 on the multiple sequence alignment. Whereas the Dotu et al. approach performs better for miRNAs and single hairpin cis-regulatory structures, RNAbound is in general superior for branched structures on multiple sequence alignments. This may be due to the fact that the weight combination ($w_{1}=2$ and $w_{2}=1$) used in the fitness function of the Dotu et al. approach was based on their benchmarking from the single hairpin structures (like miRNAs) [2], and probably they are not optimal for branched structures. However, the advantage of RNAbound is that it does not require any such weight combinations pertained to each RNA family.

## 4. Discussion

The power of multiple sequence alignments has previously been shown for accurate prediction of the secondary structures of RNA sequences, de novo finding of structured RNAs, and clustering of RNA structures. We present here that multiple sequence alignments are also in favor, compared to single sequences, for predicting the boundaries of branched structured RNAs. We show that the strength of improvement is, however, dependent on the shape of the structure, the sequence identity of the alignment, and the scoring function.

**Figure 5:**
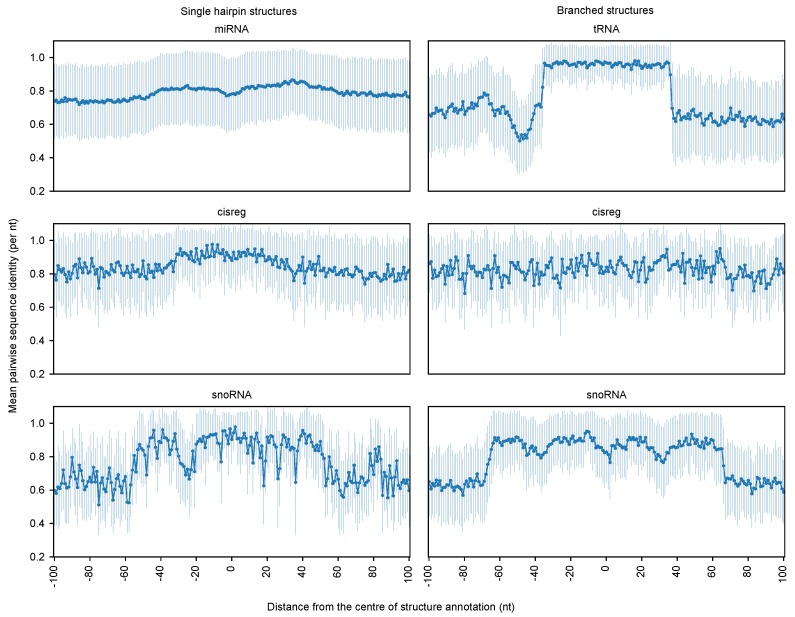
The mean pairwise sequence identity of each position along the multiple sequence alignment is shown for window size 200 nts. The *x*-axis represents each position along the multiple sequence alignment, and the *y*-axis represents the mean pairwise sequence identity computed per position in an alignment corresponding to each Rfam family. The circle indicates the mean of all mean pairwise sequence identities and vertical lines represents the standard deviation for each position on the *x*-axis. In case of alignments longer than the window size (200 nts), due to gaps introduced in the human sequence, we ignored the gapped columns for the sequence identity calculation. In addition, columns that contain greater than 25% bases as gaps are ignored in this calculation.

We used global folding of a specified window size to calculate base pairing probabilities with RNAfold and PETfold. However, a major challenge in global folding is the correct prediction of long-ranging base pairs. Given that the majority of base pairs have short base pair spans and local structure can be predicted without the stabilizing effects of long-range connections, local folding approaches could replace the global folding strategy and perhaps improve boundary estimations, e.g., Rfold [25], Raccess [26], RNAplfold [27], and LocalFold [28]. In addition, the quality of the input alignment has a large impact on the capability to predict correct structured RNA boundaries. Sequence conservation is highly correlated to the quality of sequence-based alignments. Figure 5 illustrates that on average the sequence identity drops when we move away from the annotated structured RNAs. Previous studies have shown that the structure prediction from sequence-based alignments with sequence identity below 60% is inaccurate, thus considering a lower threshold for sequence identity to compute base pair probabilities may improve the accuracy. Instead of selecting a static window size for base pairing probability calculation, an improved strategy would be to choose a dynamic window size based on the sequence conservation.

In this study, we have compared two fitness functions that are based on base pairing probabilities. Despite the similarity of the fitness functions, they outperform each other on different RNA structure families. Our method, RNAbound, does not depend on weights to its three components of the fitness function; however, we have to define the sensitivity of the scoring for low base pairing probabilities (the null model of random pairing $p_{0}$) and the size of the margin to be considered for spanning base pairs (length *f* of flanking sequences). In Supplementary Figure S1, we present the performance of different combinations of these two variables on the benchmark data set. On the multiple sequence alignments in this data set, a high null model probability gives best results for single hairpin structures, e.g., miRNAs. In contrast, for more complex structures, lower base pairing probabilities are important to consider. In addition, the length of the flanking sequences are impacting the ability to predict correct boundaries where, for single hairpin structures, the additional information of longer flanking sequences often improves the predictions. In summary, no specific combination of the two variables exist that performs best for all window sizes and all RNA structure families. In future work, the boundary predictions could be optimized by considering the shape of the predicted optimal RNA secondary structure, such as minimum-free energy or centroid structure, for setting the values of $p_{0}$ and *f*. Alternative approaches such as machine learning may further improve the boundary prediction accuracy from a global perspective when being trained by a large data set of different RNA families.

In our benchmark dataset, the boundaries of single hairpin structures are more precisely predicted from single sequences than from multiple alignments (Figure A1). However, this observation is driven by a large number of miRNAs in the dataset (Figure 2). We found that the ensemble of miRNA structure is often more diverse when calculated from an alignment compared to the folding of the well-annotated human sequence. In addition, tRNAs dominate the set of branched structures, however, at a lower extent. In the design of the benchmark dataset, the overrepresentation of miRNAs and tRNAs in the structured RNA annotation of vertebrate genomes may be overcome through the simulation and/or sampling of structured RNA families with different secondary structure shapes.

Self-contained RNA structures can occur in clusters including some small RNAs, such as tRNAs and microRNAs. Hence, the input sequence or alignment to RNAbound can comprise more than one self-contained structure. In the benchmark data set, the initial 2385 multiple sequence alignments that overlap at least one annotation originating from an Rfam seed alignment contain only around 5% that overlap (≥50 nts) with more than one annotation (127 alignments correspond to 300 Rfam annotations). Because the majority of the annotated structures are not close to another structure, we focused on the boundaries of isolated structures to test the fitness functions. In future work, not only the self-contained RNA structure of the highest score should be searched, but, instead, all non-overlapping structures of comparable scores to identify clusters. For the study sub-optimal self-contained structures, we provide a script that filters non-overlapping regions with highest fitness function scores from the RNAbound output.

Putative applications of RNAbound are in post-processing the recurrent RNA structures detected through de novo structured RNA finding approaches. Either shrinkage of boundaries of window-based approaches or expanding the boundaries of local alignment based approaches may better capture the structural features of RNAs. In addition, identifying and annotating the genomic occurrence of homologous RNA structure motifs from sets of biologically related sequences will improve our understanding of the structure-function relationship of RNAs and the molecular mechanisms underlying their regulatory features. Putative RNA structure motifs may be hidden in signals from RNA sequencing experiments that measure how RNAs interact with other molecules, such as cross-linked RNA immuno-precipitation for exploring RNA-protein interactions. Post-processing comprises RNA structure modelling, e.g., through covariance models [29], and clustering of RNA structures. Whereas structure-based RNA alignments typically identify the common structure for orthologous RNAs, clustering seeks to group paralogous RNAs based on structural similarities. For instance, the recent clustering tool DotAligner [30] searches for semi-local pairwise alignments by introducing penalty-free gaps at the sequence extremities to overcome this limitation; however, a pre-processing of more accurate structure boundaries would improve the sensitivity and specificity of the clustering.

## Figures and Tables

**Table 1:** Benchmark dataset.

RNA Family	Window = 100	150	200
miRNA	244	642	661
tRNA	185	166	127
cisreg	30	42	46
snoRNA	6	37	81
ribozyme	2	3	2
snRNA	1	3	6
others	1	6	9
lncRNA	0	0	1
Total	469	899	933

miRNA: microRNA; tRNA: transfer RNA; cisreg: cis-regulatory RNAs; snoRNA: small nucleolar RNA; snRNA: small nuclear RNA; lncRNA: long non-coding RNA.

**Table 2:** The table shows the median value of the differences between the predicted and actual boundaries of structured RNAs using RNAbound and Dotu et al. approaches. The base pair probabilities for single sequences and multiple sequence alignments were computed using RNAfold and PETfold, respectively. Only Rfam families with more than five entries are listed here.

Window	Family (*n*)	RNAbound				Dotu et al.			
		Single seq.		Multiple seq.		Single seq.		Multiple seq.	
		Left	Right	Left	Right	Left	Right	Left	Right
100	**Single hairpin structures**								
	miRNA (244)	0.0	−2.0	4.0	1.0	−2.0	−3.0	1.0	0.0
	cisreg (28)	6.5	−1.0	5.0	−2.0	−5.0	−5.0	−8.5	−15.5
	snoRNA (6)	2.0	−2.5	1.5	−6.0	−19.0	−16.0	−18.0	−17.5
	all (279)	0.0	−2.0	4.0	0.0	−2.0	−4.0	1.0	−1.0
	**Branched structures**								
	tRNA (185)	−5.0	−7.0	−5.0	−9.0	1.0	−2.0	0.0	−2.0
	all (190)	−5.0	−7.0	−5.0	−9.0	1.0	−2.0	0.0	−2.0
150	**Single hairpin structures**								
	miRNA (642)	10.0	6.0	11.0	9.0	−5.0	−6.0	−1.0	−1.5
	cisreg (36)	13.0	19.5	9.0	7.0	−0.5	−3.5	−5.0	−8.5
	snoRNA (24)	8.0	6.0	5.5	−1.5	−27.5	−29.5	7.0	3.0
	all (709)	10.0	7.0	9.0	8.0	−6.0	−7.0	−1.0	−2.0
	**Branched structures**								
	tRNA (166)	18.0	16.0	−6.0	−9.0	2.0	−1.0	0.0	−2.0
	cisreg (6)	11.5	13.5	−4.5	3.0	−0.5	−21.5	−4.5	1.0
	snoRNA (13)	0.0	−8.0	0.0	−12.0	−12.0	−71.0	−14.0	−82.0
	all (190)	14.5	14.0	−6.0	−9.5	1.0	−2.0	0.0	−3.0
200	**Single hairpin structures**								
	miRNA (661)	30.0	30.0	30.0	33.0	−6.0	−7.0	−2.0	1.0
	cisreg (37)	35.0	35.0	33.0	29.0	5.0	−4.0	−5.0	−16.0
	snoRNA (20)	24.5	8.5	12.0	5.0	−52.0	−4.5	7.0	3.0
	all (727)	30.0	29.0	29.0	32.0	−6.0	−7.0	−1.0	0.0
	**Branched structures**								
	tRNA (127)	38.0	39.0	−6.0	−9.0	2.0	−2.0	0.0	−3.0
	cisreg (9)	23.0	17.0	23.0	26.0	−5.0	3.0	16.0	26.0
	snoRNA (61)	11.0	−2.0	−7.0	−69.0	−20.0	−24.0	−18.0	−86.0
	all (206)	23.0	18.5	−6.0	−11.5	−14.5	−12.5	−14.0	−9.0

## Data Availability

The source codes of RNAbound and the benchmarking dataset are available at http://rth.dk/resources/rnabound.

## Supplementary Materials

The following are available online at http://www.mdpi.com/2073-4425/9/12/604/s1, Figure S1: Performance of RNAbound with different parameter combinations, Table S1: Filtering counts of annotated structured RNAs in benchmark dataset.

## Appendix A

**Table A1:** Summary of boundary predictions from multiple sequence alignments with RNAbound and Dotu et al. The base pair probabilities were calculated with PETfold.

Window	Family (*n*)	RNAbound				Dotu et al.			
		Left		Right		Left		Right	
		Median	std	Median	std	Median	std	Median	std
100	**Single hairpin structures**								
	miRNA (244)	4.0	12.50	1.0	14.34	1.0	16.85	0.0	14.30
	cisreg (28)	5.0	10.23	−2.0	14.83	−8.5	14.58	−15.5	18.21
	snoRNA (6)	1.5	10.57	−6.0	15.80	−18.0	15.20	−17.5	18.86
	all (279)	4.0	12.47	0.0	14.39	1.0	16.67	−1.0	14.93
	**Branched structures**								
	tRNA (185)	−5.0	11.07	−9.0	12.38	0.0	21.19	−2.0	11.17
	cisreg (2)	7.5	3.54	7.5	3.54	5.0	0.0	7.5	2.12
	ribozyme (2)	−8.0	15.56	−10.5	2.12	−25.0	35.36	−8.0	7.07
	snRNA (1)	−33.0	0.00	−12.0	0.00	−33.0	0.00	−13.0	0.00
	all (190)	−5.0	11.07	−9.0	12.41	0.0	21.23	−2.0	11.16
150	**Single hairpin structures**								
	miRNA (642)	11.0	27.29	9.0	26.62	−1.0	33.91	−1.5	29.71
	cisreg (36)	9.0	18.35	7.0	21.42	−5.0	21.14	−8.5	24.76
	snoRNA (24)	5.5	19.48	−1.5	16.01	7.0	15.46	3.0	21.32
	all (709)	9.0	26.61	8.0	26.04	−1.0	32.86	−2.0	29.17
	**Branched structures**								
	tRNA (166)	−6.0	18.34	−9.0	20.09	0.0	22.96	−2.0	14.53
	cisreg (6)	−4.5	24.70	3.0	39.39	−4.5	50.49	1.0	40.18
	snoRNA (13)	0.0	21.45	−12.0	36.90	−14.0	32.78	−82.0	44.22
	ribozyme (3)	−51.0	32.97	−14.0	15.39	−50.0	33.26	−13.0	15.28
	snRNA (3)	−17.0	15.01	−50.0	33.60	−80.0	32.75	−13.0	9.02
	all (190)	−6.0	19.23	−9.5	23.24	0.0	26.23	−3.0	21.68
200	**Single hairpin structures**								
	miRNA (661)	30.0	43.96	33.0	39.94	−2.0	49.87	1.0	45.70
	cisreg (37)	33.0	31.60	29.0	41.10	−5.0	29.04	−16.0	34.79
	snoRNA (20)	12.0	34.34	5.0	44.38	7.0	28.95	3.0	33.36
	all (727)	29.0	43.47	32.0	40.06	−1.0	45.58	0.0	45.27
	**Branched structures**								
	tRNA (127)	−6.0	30.38	−9.0	34.29	0.0	29.02	−3.0	19.67
	cisreg (9)	23.0	32.20	26.0	61.57	16.0	35.36	26	75.80
	snoRNA (61)	−7.0	36.62	−69.0	44.87	−18.0	36.99	−86.0	39.29
	ribozyme (2)	−35.0	49.50	−14.5	31.82	−31.5	44.55	−18.0	21.21
	snRNA (6)	−22.0	38.90	−48.5	29.95	−86.0	44.46	−12.0	32.94
	all (206)	−6.0	34.85	−11.5	43.77	−14.0	35.76	−9.0	40.63

**Table A2:** Summary of boundary predictions from single sequences with RNAbound and Dotu et al. The base pair probabilities were calculated from the human sequences with RNAfold.

Window	Family (*n*)	RNAbound				Dotu et al.			
		Left		Right		Left		Right	
		Median	std	Median	std	Median	std	Median	std
100	**Single hairpin structures**								
	miRNA (244)	0.0	7.35	−2.0	9.12	−2.0	10.70	−3.0	10.08
	cisreg (28)	6.5	12.01	−1.0	12.57	−5.0	12.12	−5.0	17.51
	snoRNA (6)	2.0	17.71	−2.5	21.92	−19.0	12.04	−16.0	15.95
	all (279)	0.0	8.63	−2.0	10.00	−2.0	11.11	−4.0	11.19
	**Branched structures**								
	tRNA (185)	−5.0	11.53	−7.0	11.37	1.0	15.90	−2.0	14.47
	cisreg (2)	−5.0	15.56	−10.5	21.92	−5.5	7.78	−9.5	17.68
	ribozyme (2)	−1.5	6.36	−5.5	0.71	−23.0	35.36	−6.0	9.90
	snRNA (1)	−33.0	0.00	−2.0	0.00	−33.0	0.00	−4.0	0.00
	all (190)	−5.0	11.48	−7.0	11.34	1.0	16.06	−2.0	14.41
150	**Single hairpin structures**								
	miRNA (642)	10.0	17.39	6.0	18.57	−5.0	19.90	−6.0	18.42
	cisreg (36)	13.0	24.99	19.5	21.81	−0.5	21.95	−3.5	23.33
	snoRNA (24)	8.0	12.44	6.0	19.65	−27.5	26.06	−29.5	24.07
	all (709)	10.0	17.64	7.0	18.91	−6.0	20.62	−7.0	19.21
	**Branched structures**								
	tRNA (166)	18.0	21.07	16.0	22.31	2.0	30.55	−1.0	28.52
	cisreg (6)	11.5	35.74	13.5	22.69	−0.5	32.62	−21.5	46.07
	snoRNA (13)	0.0	35.47	−8.0	30.95	−12.0	34.08	−71.0	40.03
	ribozyme (3)	2.0	40.81	1.0	25.17	−48.0	30.75	−13.0	15.01
	snRNA (3)	7.0	8.08	−22.0	40.93	−81.0	32.91	−4.0	6.82
	all (190)	14.5	24.63	14.0	25.12	1.0	32.05	−2.0	31.34
200	**Single hairpin structures**								
	miRNA (661)	30.0	29.63	30.0	29.15	−6.0	29.80	−7.0	29.62
	cisreg (37)	35.0	32.03	35.0	35.66	5.0	38.48	−4.0	41.38
	snoRNA (20)	24.5	19.53	8.5	18.64	−52.0	48.98	−4.5	31.54
	all (727)	30.0	29.63	29.0	29.45	−6.0	32.30	−7.0	30.54
	**Branched structures**								
	tRNA (127)	38.0	31.42	39.0	33.16	2.0	47.27	−2.0	43.57
	cisreg (9)	23.0	54.76	17.0	23.21	−5.0	45.72	−2.0	43.57
	snoRNA (61)	11.0	36.62	−2.0	39.95	−20.0	44.11	−24.0	40.40
	ribozyme (2)	15.5	19.09	13.0	15.56	−17.0	52.33	−51.0	31.11
	snRNA (6)	−3.0	39.36	−6.5	35.15	−94.5	14.96	−8.5	23.94
	all (206)	23.0	37.03	18.5	39.14	−14.5	50.82	−12.5	46.18
