# Supplementary material for: Multiple Sequence Alignments Enhance Boundary Definition of RNA Structures
Source: Genes (Basel). 2018 Dec 4;9(12):604. doi: 10.3390/genes9120604 (PMC6315940; doi:10.3390/genes9120604)
Supplement: Supplementary file 1 [file genes-09-00604-s001.zip › suppl_Figure_S1.pdf]

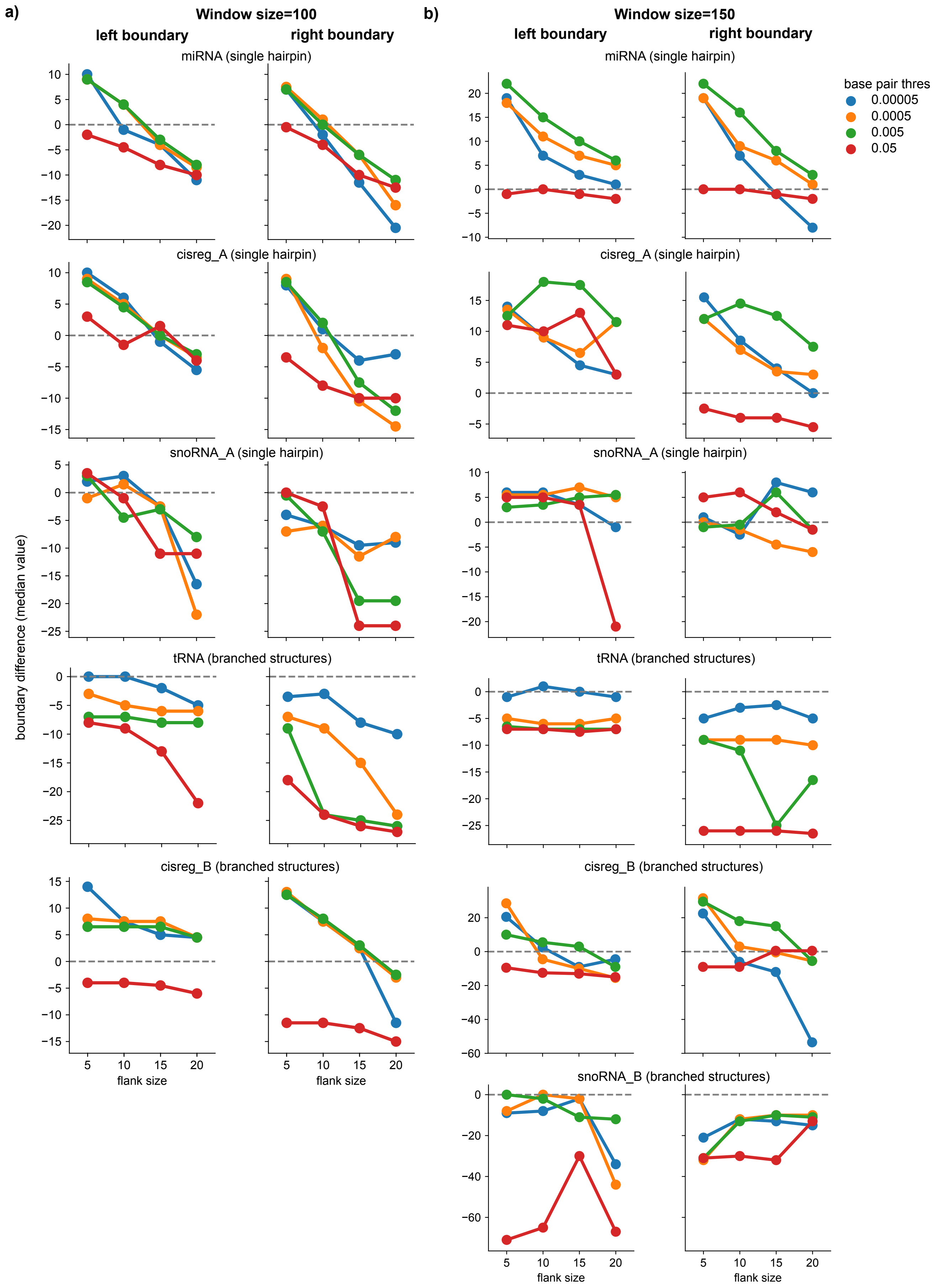
a)
Window size=100
left boundary
right boundary
b)
Window size=150
left boundary
right boundary
miRNA (single hairpin)
cisreg_A (single hairpin)
snoRNA_A (single hairpin)
tRNA (branched structures)
cisreg_B (branched structures)
snoRNA_B (branched structures)
boundary difference (median value)
flank size
base pair thres
0.00005
0.0005
0.005
0.05

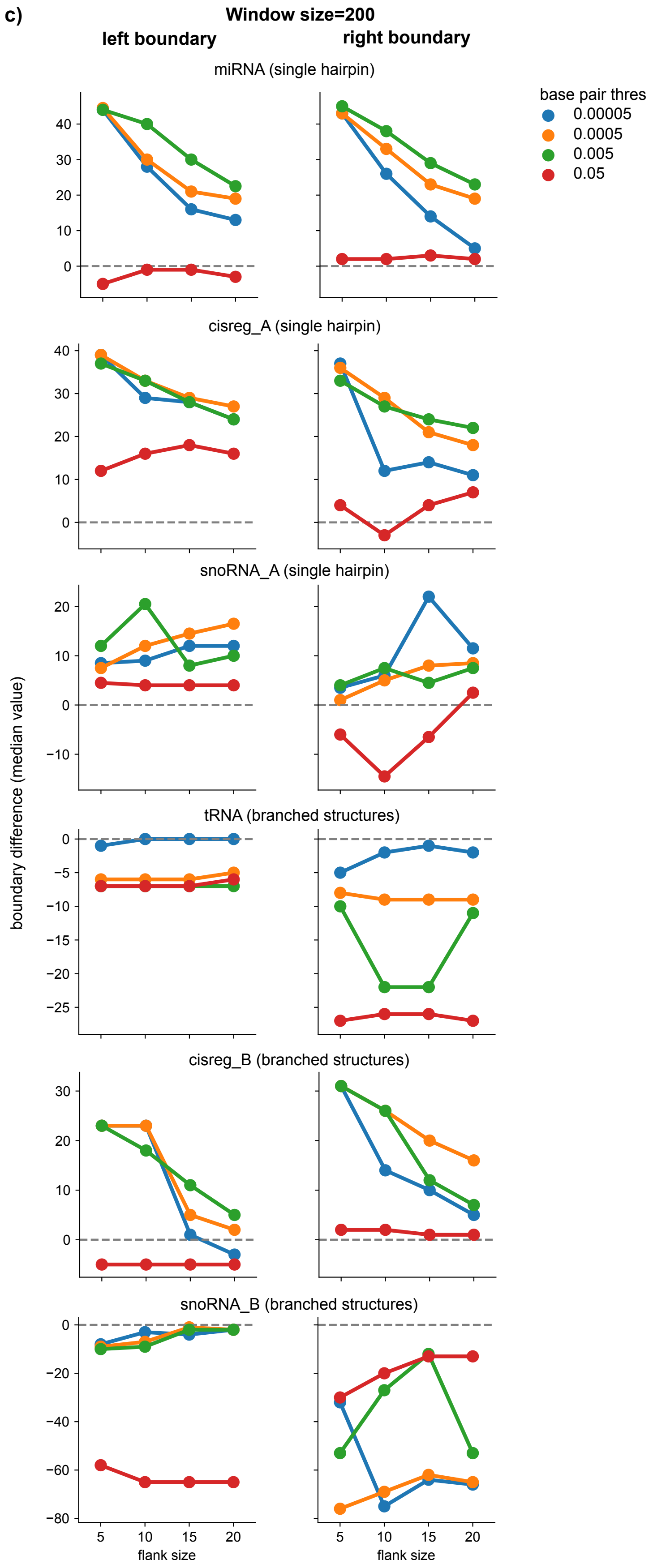

Figure S1: RNAbound results of different parameter combinations, flank size ($f = 5, 10, 15, 20$) and base pairing probability threshold ($p_0 = 0.00005, 0.0005, 0.005, 0.05$), tested on window sizes 100 (a), 150 (b), and 200 (c) across Rfam families. In each panel, the left and right column represents the results of left and right boundary differences (between predicted and actual boundaries) for each Rfam family (row wise). The x-axis represents the different flank sizes, and the y-axis represents the median value of boundary differences.
